# Stock Dynamics of Female Red King Crab in a Small Bay of the Barents Sea in Relation to Environmental Factors

**DOI:** 10.3390/ani15010099

**Published:** 2025-01-04

**Authors:** Alexander G. Dvoretsky, Vladimir G. Dvoretsky

**Affiliations:** Murmansk Marine Biological Institute of the Russian Academy of Sciences (MMBI RAS), 183038 Murmansk, Russia; ag-dvoretsky@yandex.ru

**Keywords:** red king crab, females, Barents Sea, temperature conditions, climate, abundance

## Abstract

Stock–recruitment relationships depend on the total abundance of females, their fecundity, and patterns of their maturation. However, the effects of climatic conditions on the abundance, biomass, and mean weight of female red king crabs, *Paralithodes camtschaticus*, from the introduced population (Barents Sea) have not yet been studied. For this reason, we analyzed long-term fluctuations in stock indices and the average weight of an individual crab in a small bay of the Barents Sea and related these parameters to the dynamics of temperature conditions (temperature in January–December, mean yearly temperature, and temperature anomaly) in the sea. The average weight of a crab at age 6–9 had strong negative correlations with water temperature at lags 8 and 9, indicating faster female maturation in warm periods. Positive relationships were registered between temperature and stock indices for 15–19-year-old females at lag 4 and for 10–14-year-old females at lag 10, supporting the idea of higher survival rates of juveniles and their rapid development being a response to a pool of warm waters. Both redundancy and correlation analyses revealed seawater temperatures in June–August being the most important predictors of female abundance and biomass, indicating that favorable temperature conditions in the first 3 months of crab benthic life result in high survivorship rates for red king crabs.

## Simple Summary

The current state of knowledge regarding the impact of climate on the number, weight, and size of female red king crabs in the Barents Sea is insufficient for drawing any conclusions. In this study, we examined the relationship between long-term fluctuations in female stock indices and the average weight of an individual crab, as well as temperature conditions. Our analysis demonstrated a robust correlation between these variables at varying time lags (6–10 years), supporting the hypothesis that female maturation occurs more rapidly during periods of elevated temperatures. Our findings indicate that warmer water conditions are conducive to the survival and growth of young crabs. The most significant factors influencing female abundance and biomass are seawater temperatures between June and August. Our findings could prove valuable for fishery managers in anticipating periods of high crab productivity and abundance, as well as for coastal managers in predicting abundance fluctuations of red king crab in the Barents Sea.

## 1. Introduction

The red king crab, *Paralithodes camtschaticus* (Tilesius, 1815), is an important species in terms of its commercial value and the ecological role it plays in benthic communities throughout its native areas in the western and eastern Bering Sea, USA, and the Sea of Okhotsk, Russia [1]. The success of red king fisheries in native habitats initiated Soviet scientists to introduce this lithodid crab into the Barents Sea. Although this region is known to have high productivity owing to interactions between cold Arctic and warm Atlantic waters and a wide range of environmental conditions [2,3,4,5], there are no native commercially important crab species here. The transoceanic introduction experiment conducted in the 1960s was successful, and the establishment of a new self-sustaining population of red king crab in the Barents Sea was reported in the mid-1990s. For this reason, a Russian–Norwegian joint research fishery for *P. camtschaticus* occurred from 1994 to 2001 [6]. Commercial fisheries were opened in 2002 in Norwegian and in 2004 in Russian waters of the Barents Sea [1]. The red king crab supports a viable fishery with annual stocks (landings) accounting for 199, 192, and 159 thousand metric tons (11,629, 12,529, and 10,420 t) in 2021, 2022, and 2023, respectively [7,8,9].

The population dynamics of the Barents Sea red king crab have been extensively studied, owing to the species’ invasive status and high commercial importance [1,10,11,12,13,14,15,16,17]. Many factors affect the development and distribution of crustaceans [18]. Among these, climatic fluctuations play an important role in the functioning of benthic communities in the Arctic regions [19,20,21,22,23]. Some studies indicate that the biomass of the Barents Sea benthos may fluctuate considerably in response to large-scale climatic forcing [24,25,26], providing evidence that both abundance and biomass of the whole bottom community and/or its members or groups may be used as indicators of climate change [5]. Recent investigations have documented pronounced climatic shifts in the Arctic seas with a warming trend starting in the early 2000s [27,28,29]. Climatic variations in the Barents Sea can be detected as a series of temperature anomalies along standard transects of the sea [30,31].

The response of commercially important species to changing climate may vary widely because of exploitation pressure and other direct and indirect effects of environmental variations [32,33]. Recently, we analyzed inter-annual fluctuations in the total number of recruits and commercially sized male red king crabs and found some correlations between these indices and environmental factors, such as water temperature and the North Atlantic oscillation and Arctic oscillation, with time lags [34]. We concluded that the abundance of males is a poor indicator of climate shift because of fishing pressure on this group. In contrast, both the abundance and biomass of juvenile red king crabs have been shown to relate to environmental factors such as water temperature and temperature anomalies, as well as to cod predation. These correlations can vary significantly depending on the age group of juvenile red king crabs [21]. At the same time, the relationships between environmental factors and stock indices of adult female red king crabs in the Barents Sea are far less evident. Females are considered to be the most important functional groups in each red king crab population because their number and individual fecundity directly affect the number of released larvae, the number of juvenile crabs, and, therefore, the commercial stock in the area [35].

In the coastal Barents Sea, the abundance of egg-bearing females in some years may reach 19,000,000 individuals, with mean catch per unit efforts (CPUEs) for these females ranging from 1.9 to 7.3 individuals per pot [16]. Although time-series analysis has demonstrated that the introduction of red king crab and their subsequent population growth have not adversely affected local fish and shellfish stocks [36], negative consequences for coastal benthic communities associated with increased crab abundance have been observed. These consequences include a reduction in species diversity, habitat disturbance, and a simplification of benthic community structure [37,38,39,40,41,42].

Thus, knowledge about relationships between environmental drivers and stock indices of adult female red king crabs could have important implications, not only for crab fishery management, but also for the prediction of possible changes in benthic communities associated with feeding activity of the crabs and their competition for food with native inhabitants. For this reason, the aim of our paper was to investigate the effect of temperature conditions on the abundance characteristics of female red king crabs in a typical bay of the Barents Sea.

## 2. Material and Methods

### 2.1. Red King Crab Stock Indices

Red king crabs were caught by divers at depths of 8–40 m in Dalnezelenetskaya Bay in July–August, 2003–2017. A detailed description of the study area is presented in previous papers [43,44,45].

Each crab was sexed and weighed and the carapace length (CL, the straight-line distance across the carapace from the posterior margin of the right eye orbit to the medial-posterior margin of the carapace) was measured using calipers. In our study, we considered adult female red king crabs. The crabs were divided into three age groups: 6–9-year-olds (95–125 mm CL), 10–14-year-olds (126–150 mm CL), and 15–19-year-olds (151–170 mm CL), according to published size-at-age data [14,35,46,47].

The area of the bay was divided into several parts with the same types of biological communities (benthic organisms living on hard and soft grounds, kelps, and sand). A standardized transect grid comprising 10 to 25 transects was established to encompass a diverse range of depths (from 3 to 42 m) and various types of benthic communities [12,21]. When duplicate transects were analyzed, the obtained values were averaged to ensure reliable representations of the data. The number of crabs and their biomass on each transect line (observation width: 15 m) were used to estimate the total abundance (the total number of crabs) and the total biomass using a spline approximation method (the approximate representation of a function in a given class from incomplete information using splines) with the Chartmaster Software Tool ver. 1.0 [48]. In addition to the stock indices, the analyses were also applied for averaged individual weights (g) of different aged crabs.

### 2.2. Environmental Data

It is accepted that climatic conditions of the southern Barents Sea are determined by temperature characteristics of Murmansk coastal waters (stations 1–3 of the standard transect called “Kola Section” 33°30′ E, 69°–78° N, 0–200 m depth). Mean seawater temperature in the Kola Section (averaged month values for each year and the averaged year value for 50–200 m depths, which reflects near-bottom temperatures) in 1984–2017 and data on temperature anomaly in the section were derived from a long-term dataset collected by Polar Research Institute of Marine Fisheries and Oceanography (www.pinro.ru accessed on 20 March 2018). The calculation of anomaly values involved the subtraction of the mean from 1951 to 2017 from the original data and dividing the result by the standard deviation. Initially, we also tested the North Atlantic oscillation indices and salinity data as environmental predictors of the red king crab stock indices, but these factors had no effects on *P. camtschaticus* females and were excluded from further consideration.

### 2.3. Data Analysis

Firstly, we calculated simple pairwise correlations (Pearson’s correlation coefficient) between crab abundance indices and environmental factors. We used both data for the current year and data with time lags of up to 19 years according to the maximum age of a female crab in our study. The normality and homogeneity of variances were tested before performing the statistical analyses. The assumption of homogeneity of variance was verified using Levene’s test and the assumption of normality was verified using the Shapiro–Wilk test. The Benjamini–Hochberg procedure was used to correct *p*-values for multiple testing [49]. In each case, the total number of tests was 14 and a false discovery rate was set at 10%. Since simple correlation overstates the significance of associations in the case of significant autocorrelation in time-series data [50], we tested the data for temporal autocorrelations using the Box–Pierce test and found that the *p*-values were higher than the critical value of 0.05.

Secondly, to analyze the complex factor loadings and for visualization of our results, we used multivariate techniques. The influence of water temperature between 50 and 200 m depth on the biomass of females at different ages was examined using redundancy analysis (RDA). Biomass was used as a metric instead of abundance because this parameter explained a higher proportion of the total variation. RDA is an analytical tool that explicitly models response variables as a function of explanatory variables [51]. This approach can be applied to assess the effects of environmental variables on the stock characteristics of red king crabs. This method was used because a preliminary detrended correspondence analysis showed that the lengths of gradient were all less than 3 [52]. The graphical output of RDA consists of a simple biplot showing the associations between the quantitative explanatory variables and the response variables. This analysis was applied only to the dataset for which the correlation analyses indicated significant results. Prior to RDA, quantitative data were log-transformed.

Correlation testing and redundancy analyses were carried out with the PAST 3.26 and CANOCO 4.5 software packages.

## 3. Results

### 3.1. Environmental Conditions

**Figure 1:**
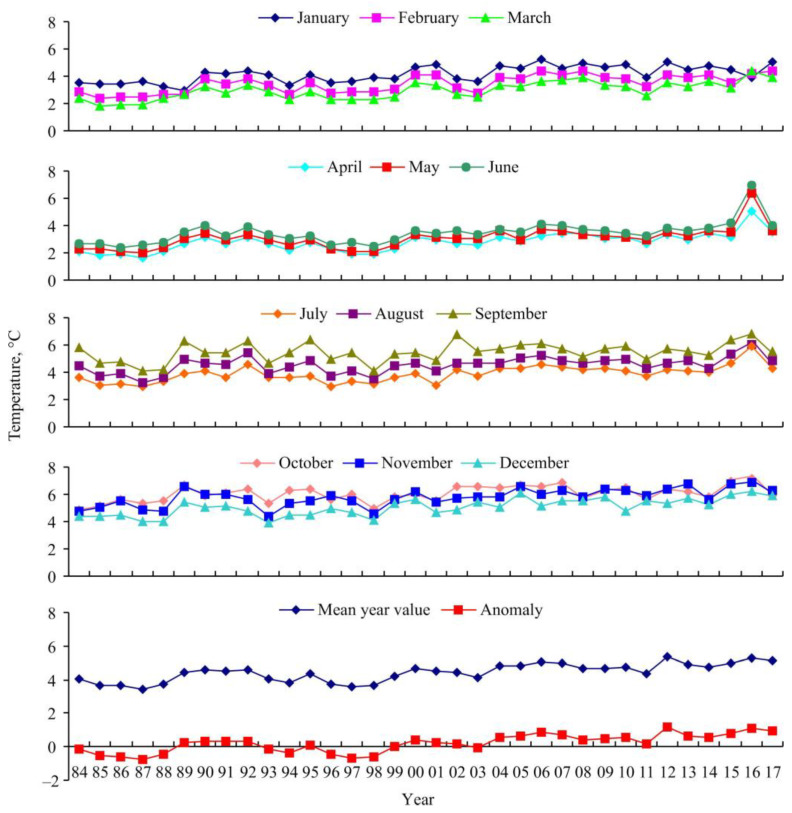
Near-bottom temperature fluctuations in the coastal zone of the Barents Sea (stations 1–3 of the Kola section at 50–200 m depth) in 1984–2017.

During the period of 1984–2017, temperature conditions varied strongly in the southern Barents Sea. Monthly and mean temperature values as well as temperature anomalies are presented in Figure 1.

**Figure 2:**
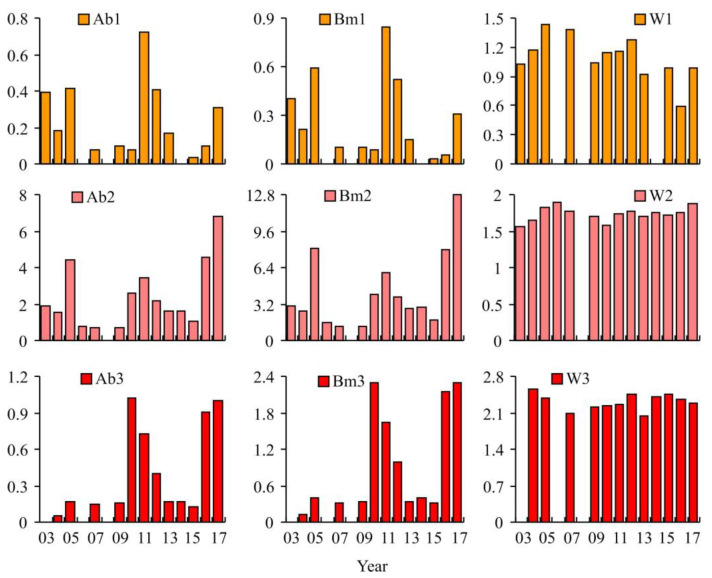
Variations in female red king crab stock indices and mean weight over 2003–2017. Ab—abundance (thousand crabs), Bm—biomass (metric tons), W—weight (kg). 1—aged 6–9 years, 2—aged 10–14 years, 3—aged 15–19 years.

March and April were the coldest months, while October and November were the warmest, at a depth of 50–200 m. A warming trend in the Barents Sea began to be registered in 1989 when negative temperature anomalies were reversed and the mean temperature increased by 1 °C in comparison to previous years. At the beginning of the twenty-first century, the advection of the North Atlantic current continued to increase. In the Barents Sea, the warm temperature anomaly peaked (0.86) in 2006, followed by a decrease in the next years (Figure 2).

A second peak (1.14) was registered in 2012. During the subsequent years, water temperature anomalies remained high (0.52–1.1). The highest levels of seawater temperature in the Kola section were found in 2006, 2012, 2016, and 2017, reaching 5.08, 5.36, 5.32, and 5.15 °C, respectively.

### 3.2. Red King Crab Stock Indices

During the study period, the 6–9-year-old group had the lowest abundance among adult female red king crabs. The maximum abundance of 725 crabs (biomass 0.84 t) was registered in 2010, while intermediate numbers of 400 crabs (0.4–0.6 t) were found in 2003, 2005, and 2011. The mean weight increased from 1.03 kg in 2003 to a maximum of 1.43 in 2005. A decreasing trend was registered in subsequent years, with a minimum value of 0.59 kg found in 2016.

A total of three peaks were registered in the abundance and biomass of *P. camtschaticus* at ages 10–14. These peaks occurred almost every 6 years across the study period: in 2005 (4415 crabs, 8.05 t), in 2011 (3460 crabs, 5.99 t), and in 2017 (6835 crabs, 12.78 t). The mean weight of a female increased from 1.57 kg in 2003 to 1.90 kg in 2006, then decreased to 1.58 kg in 2010, before increasing again to 1.87 kg in 2017.

The abundance and biomass of 15–19-year-old females were the similar between 2005 and 2009 (150–170 crabs, 0.35–0.41 t). A sharp increase in these indices was observed in 2010 (1030 crabs, 2.29 t). Over the next 3 years, the parameters decreased to the initial levels and increased again to 900–1000 crabs or 2.15–2.18 t in 2016–2017. The mean weight of a crab in this group decreased from 2.56 kg in 2004 to 2.10 in 2007. A smooth increase was observed in the next 4 years. In 2012–2015, mean weights were stable (2.40–2.45 kg) but a lower value (2.04 kg) was observed in 2013.

### 3.3. Relationships Between Crab Abundance and Environmental Variables

In the case of the 6–9-year-old group, correlation analyses indicated no significant associations between stock indices and environmental variables, while strong negative relationships were registered for the average weight of a crab from this group at time lags 8 and 9 years (Table 1).

In contrast, the abundance and biomass of females at ages 10–14 had strong positive correlations with seawater temperature in the period from January to August and in October, as well as the average annual temperature and temperature anomaly at lag 10. Strong relationships were found between pairs of stock parameters for 15–19-year-old females and environmental variables. Both abundance and biomass showed positive associations with seawater temperature from February to June, mean yearly temperature, and its anomaly at lag 4. Similar patterns were registered for the abundance and biomass of this age group at lag 10 but, in this case, significant correlations were also found for abundance in January and for biomass in January, July, and August (Table 1).

**Figure 3:**
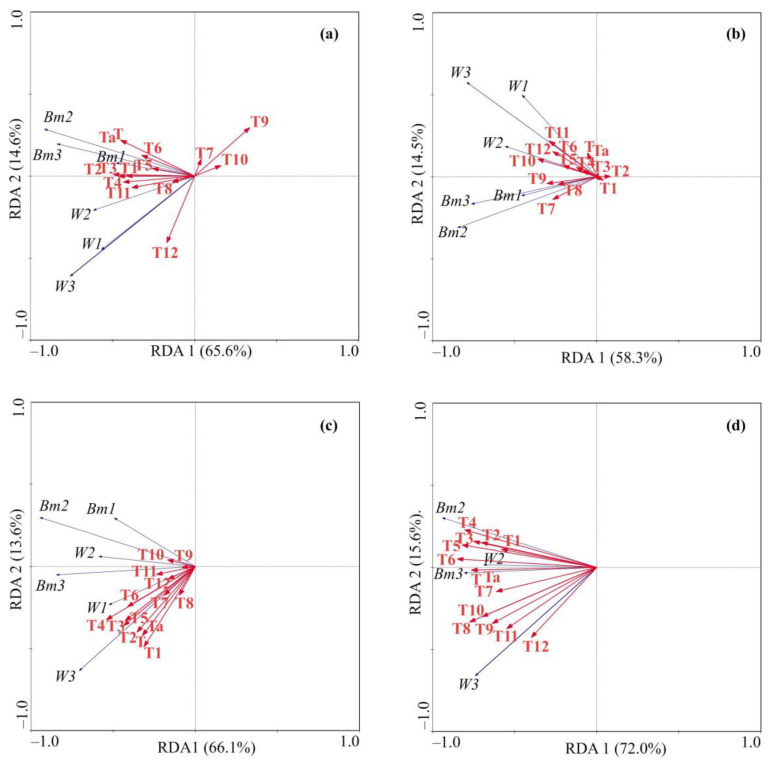
Redundancy analysis ordination plots showing female red king crab characteristics in relation to temperature variables in Dalnezelenetskaya Bay at lag 4 (**a**), lag 8 (**b**), lag 9 (**c**), and lag 10 (**d**). T1–T12—water temperature at stations 1–3 of the Kola section at 50–200 m depth in January–December, T—averaged year water temperature, Ta—temperature anomaly. Crab indices: Bm—biomass, W—weight. Age groups: 1—aged 6–9, 2—aged 10–14, 3—aged 15–19.

In general, multivariate RDA confirmed the results of simple correlation analyses. For the lag 4 dataset, the largest portion of environmental variables was negatively associated with the first RDA axis (Figure 3a), explaining 65.6% of the total variation in the crab indices. Along Axis 2, the biomass data were positively scaled with the majority of environmental variables (Figure 3a). This axis explained 14.6% of the total variation. Using the data obtained for environmental variables and red king crab indices at lag 8 as input parameters for RDA, we found that the first two axes accounted for 72.8% of the total variance of the crab parameters. The first axis was negatively correlated with almost all variables, while the second axis was positively correlated with the weight and negatively correlated with the biomass data (Figure 3b). The forward selection of environmental factors with Monte Carlo permutation tests reveals mean yearly temperature as the main factor contributing to the observed variability in the weight data, explaining 19% of the total variation.

In the case of the lag 9 dataset, the first two RDA axes explained 79.7% of the total variance in the crab indices and environmental factors. The RDA showed that the most important predictors were seawater temperature in April and the average water temperature, explaining 22% (F = 3.63, *p* = 0.033) and 18% (F = 3.64, *p* = 0.021) of variability in the average crab weight, respectively. These factors were positively scaled mainly with the data for 6–9-year-old crabs (Figure 3c).

RDA applied to the lag 10 dataset showed that environmental variables were negatively associated with the first axis and positively with the second axis, explaining 72.7% and 16.0% of the data variation, respectively. Based on RDA results, seawater temperatures demonstrated strong associations with some crab indices (Figure 3d). Significant factor contributions were found for June (F = 14.20, *p* = 0.001), July (F = 2.71, *p* = 0.049), and August (F = 3.08, *p* = 0.017). These factors explained 52%, 9%, and 9% of the total variation, respectively.

## 4. Discussion

Previous research has indicated that the fluctuations in the abundance of both fish and crabs in Dalnezelenetskaya Bay accurately reflect the broader ecological patterns observed in the Barents Sea [14,21,53]. Consequently, this area can be regarded as a reference site for studying the impacts of environmental factors on specific species, including commercially significant red king crabs and gadid fish. We found that the abundance and biomass of *P. camtschaticus* females varied considerably among age groups. The youngest female crabs were less abundant than older specimens. This pattern seems to reflect the higher mortality rate of 6–9-year-old crabs, owing to their higher vulnerability to predators. In our previous paper, we showed that smaller *P. camtschaticus* juveniles are consumed by the Northeast Arctic cod *Gadus morhua*, a major red king crab predator in the Barents Sea, in substantially higher amounts than larger king crabs [10,21]. Thus, smaller ovigerous females seem to be more vulnerable to predators’ attacks including cod and marine mammals. It is important to emphasize that smaller female red king crabs exhibit a tendency to remain within a specific area throughout the year [11]. Therefore, the observed fluctuations are unlikely to be related to changes in migratory patterns. In addition, smaller females are less competitive with their larger conspecifics, both females and males. Competition between different-sized red king crabs is supported by the fact that no aggregations of mixed age classes were found during the study period in the area. This result is in contrast to the patterns registered for *P. camtschaticus* in Alaskan waters, where ovigerous female red king crabs exhibited “podding” behavior, similar to that reported for juveniles [54]. It is important to note that, while larger female red king crabs exhibit seasonal migrations, departing the area in autumn and returning in spring for egg release and spawning [11], there is currently no evidence to support significant variations in the migratory patterns and routes of female red king crabs in the study area [14]. Consequently, the annual influx of newly migrated crabs reflects the general population fluctuations driven by natural mortality and recruitment, both of which are influenced by temperature fluctuations and other temperature-related indices, such as the biomass of predators [21]. It is also not surprising, that the oldest females were less abundant than the females from the 10–14-year age group. Aging as an animal means degradation of all physiological functions, a decrease in mobility, high vulnerability to disease and parasites, and, finally, higher mortality [11,32] [Harman, 2003]. Illegal fishing could also be considered as a factor responsible for the higher mortality of older females because larger and heavier crabs are preferable for fishermen than smaller specimens [14,55]. It is noteworthy that within the studied area, significant fishery activities were not observed throughout the study period. The interannual fluctuations observed were primarily attributed to variations in the local temperature regime. Since 10–14-year-old females predominated in terms of their abundance and biomass, natural fluctuations in their stock indices were clearly expressed. We found that abundant cohorts occur every 6 years. This pattern reflects red king crab recruitment oscillations and recruit–stock relationships in this species and is in good accordance with the patterns reported in the Sea of Okhotsk [56].

The effects of climate shifts on marine communities, in general, and their components, in particular, have been the subject of many studies [57,58,59,60]. Responses of benthic assemblages to cooling or warming have been shown to depend on their origin and biogeographical status. For example, in cold years, the abundance of true arctic species increases, while the abundance of boreal and arctic-boreal species decreases [5,24,25]. So, after cooling, some cold-water polychaetes have been shown to shift their distribution to the northern areas of the Barents Sea while, after warming, such species disappeared from the coastal areas where they were abundant in cold and normal years. As a rule, the responses of benthic animals, especially long-lived, to environmental forcing factors are registered with time lags, owing to a cascading effect from the physical stimulus to benthic biological response, which is presumably mediated through constraints on primary production [58,61]. Red king crabs are also affected by climatic conditions [21,34] and, in general, their responses to a changing environment are similar to those observed for other benthic species [62,63,64].

In Dalnezelenetskaya Bay, a strong positive relationship was observed between temperature conditions and the total number of 15–19-year-old females at lag 4. This result, in our opinion, reflects direct temperature effects on the biomass of benthic animals consumed by *P. camtschaticus*. In the coastal areas of the Barents Sea, echinoderms (sea urchins, sea stars, and brittle stars) play an important role in the diet of adult *P. camtschaticus* because they provide red king crabs with calcium, which is crucial for shell hardening after the crabs molt [37,65,66]. According to the data reported by Frolova et al. [25], the biomasses of echinoderms are directly linked to temperature conditions, but an increase in these parameters as a response to climate forcing is registered at lag 3 for brittle stars and lag 4 for sea stars, which can explain the pattern observed for our lag 4 dataset.

We found negative relationships between the average weight of a female crab at ages 6–9 and environmental conditions at lags 8 and 9. At the same time, we found no negative associations between abundance and water temperatures. This result means that, in warm years, the cohorts of 0–1-year-old crabs are composed of smaller and lighter specimens than in cold and normal years. Growth and molting in red king crabs are temperature-dependent [67], and the maturation of female red king crabs in warmer waters is faster than in colder waters; therefore, females living in more favorable conditions reach sexual maturity earlier than the same-aged individuals from colder environments [68]. In addition, a predator-induced mortality rate in smaller juveniles (age 0–2) is lower than in larger individuals (age 0–5) due to differences between their behavior patterns which are closely related to their vulnerability to predators [21].

The most obvious results were obtained for the abundance and biomass of crabs at ages 15–19 and, especially, 10–14. Strong positive correlations were found for the lag 10 dataset. Oscillations in the climate, coupled with shifts in advection, are likely to affect the survival rates of pelagic larvae and reproductive patterns of red king crab populations [12,69,70]. In cold periods, when the ice cover in the Barents Sea is extended, the period available for the growth of primary producers (microalgae) tends to be shorter [71]. Consequently, cold periods lead to decreasing biomasses of phytoplankton, their predators, and dissolved organic carbon [72]. The pool of food sources for benthic animals, including prey for red king crabs, in such periods is smaller than in warm periods. Furthermore, a complex of biological processes at shallow water sites of the Barents Sea plays a pivotal role in the life cycle of *P. camtschaticus* because adult crabs use the coastal zone for spawning, and juveniles spend their first years of life exclusively at depths not exceeding 100 m [12,35]. Suspended sediments from upland areas are delivered into the nearshore zone of the Barents Sea by river discharge and coastal erosion [73]. These processes are known to have a close association with climate change, which is expressed in warmer years [74]. Thus, an increase in the total terrestrial inputs of particulate organic carbon leads to the increased productivity of benthic marine communities in the Arctic [75,76,77], including red king crab abundance [34,78]. All these reasons can explain why the abundance of female red king crabs tended to increase after warming and decrease after cooling. Similar patterns have been recorded for stocks of commercial male red king crabs both in their native area (Gulf of Alaska and the eastern Bering Sea, [79]) and in the area of introduction [34]. In addition to abundance, changes in climatic conditions may affect the distribution of female red king crabs. For example, in Bristol Bay, a pool of very cold water led to a shift in the center of abundance from the south-west part to the center of the area [80].

Both simple correlation analyses and RDA applied to the lag 10 dataset indicated that temperature conditions in the summer period are the most important predictors of female abundance (age 10–19) in the coastal zone of the Barents Sea. It is known that the reproductive cycle of *P. camtschaticus* lasts for 11–11.5 months, with a spawning peak in April, a peak of larval release during May–April, and a peak of larval settlement, as well as a transition from a swimming planktonic larva to a crawling benthic juvenile [11]. Since red king crabs have a relatively narrow temperature tolerance [67,81], their survival rates at the first 3 months of life on the seafloor have close associations with temperature and temperature-related conditions such as feeding habits. Other important factors are the availability and characteristics of suitable benthic habitats. During this critical period when they are highly vulnerable, post-settlement red king crabs are also exposed to a new suite of predators and competitors [82,83]. Thus, the temperature regime in coastal waters of the Kola peninsula directly influences mortality, recruitment patterns [21], and, hence, population abundance of red king crabs [70].

Fishing activities exert a significant impact on stock biomass, potentially leading to adverse effects on recruitment when stock biomass levels are critically depleted. This phenomenon is particularly relevant for the male component of a crab population in male-only fisheries [84]. From this perspective, the abundance of females becomes a more suitable metric for examining the relationship between stock dynamics and climatic variations. As the modeling of stock dynamics in the Barents Sea incorporates female abundance of the red king crab, our data hold significant implications for fishery management strategies in this region. Historically, the inability to sustain productive crab fisheries over extended periods has necessitated the adoption of progressively more conservative management approaches [9]. Consequently, this has precipitated dramatic declines in red king crab abundance, both within their native distribution areas and in the Barents Sea [1,12,55,85]. However, in the latter region, timely conservation measures have facilitated the recovery of the stock. Our study has identified time lags of 4 and 10 years in the response of female red king crabs to changes in temperature regimes. These findings provide a crucial foundation for more accurate forecasting of female abundance, thereby informing and refining the management practices for the sustainable exploitation and conservation of the red king crab stock in the Barents Sea.

## 5. Conclusions

The abundance and biomass of female red king crabs, as well as their mean weight, are closely associated with temperature conditions. Climate forcing leads to a decrease in mortality and provides favorable food conditions for juvenile red king crabs. As a result, a peak in abundance is expected after 10 years of population development. This pattern is also relevant for adult males and, therefore, can be used for the prediction of the total population number, abundance of recruits, and the commercial stock of *P. camtschaticus* in the Barents Sea. Taking into account that fluctuations in abundance are accompanied by fluctuations in distribution, our data may be useful for scientists and managers involved in tracking the range expansion of *P. camtschaticus* in the Barents Sea and evaluating its impact on local benthic assemblages. Although red king crabs are well adapted to the new habitat conditions and their impact on the major fish stocks is considered to be neutral, some authors suggest that there are negative consequences caused by these invaders on benthic fauna at several coastal sites and on the recruitment of non-commercial fish. Indirect effects of crab predation have been shown to include a positive cascade effect on macroalgae due to predation on herbivorous sea urchins, a negative effect on benthic-feeding birds, and the dispersion of crab-associated fish leeches that can increase transmission of trypanosomes to cod. Because the increased abundance and range expansion of red king crabs into new areas can be expected as a response to climate forcing, the managers of alien species should be prepared to undertake appropriate steps and measures to prevent negative scenarios.

## Figures and Tables

**Table 1:** Correlation coefficients (r) between red king crab abundance, stock and weight characteristics, and environmental conditions in the Barents Sea in 2003–2017.

Parameter	Ab1	Ab2	Ab3	Bm1	Bm2	Bm3	W1	W2	W3
	Lag 4								
T1	0.104	0.333	0.501	0.127	0.312	0.508	−0.211	−0.154	0.261
T2	0.281	0.408	**0.524**	0.298	0.388	**0.532**	−0.055	−0.108	0.359
T3	0.360	0.366	**0.514**	0.375	0.349	**0.522**	0.010	−0.067	0.283
T4	0.242	0.306	**0.546**	0.255	0.293	**0.556**	−0.142	0.043	0.256
T5	0.089	0.203	**0.556**	0.102	0.187	**0.554**	0.000	0.002	0.018
T6	0.168	0.260	**0.643**	0.167	0.245	**0.637**	0.107	0.063	−0.130
T7	−0.151	−0.116	0.435	−0.223	−0.130	0.425	−0.292	−0.152	−0.257
T8	−0.149	−0.014	0.466	−0.217	−0.031	0.447	−0.330	−0.220	−0.445
T9	−0.427	−0.280	0.041	−0.464	−0.278	0.027	−0.463	0.161	−0.490
T10	−0.173	−0.184	0.248	−0.220	−0.186	0.228	−0.261	0.060	−0.602
T11	−0.042	0.348	0.476	−0.147	0.355	0.473	−0.471	0.065	−0.246
T12	0.129	0.012	0.141	0.039	0.013	0.137	0.187	−0.224	−0.261
T	0.024	0.444	**0.739**	−0.029	0.423	**0.743**	−0.373	−0.059	0.024
Ta	0.024	0.444	**0.739**	−0.029	0.423	**0.743**	−0.373	−0.059	0.024
	Lag 8								
T1	−0.380	0.003	0.076	−0.423	0.022	0.094	**−0.717**	0.176	0.057
T2	−0.408	−0.022	0.063	−0.458	−0.010	0.081	**−0.701**	0.022	0.061
T3	−0.336	0.013	0.161	−0.393	0.022	0.178	**−0.666**	−0.007	0.078
T4	−0.194	0.050	0.267	−0.273	0.045	0.282	**−0.649**	−0.192	0.109
T5	−0.070	0.043	0.310	−0.128	0.038	0.322	**−0.593**	−0.173	0.105
T6	−0.120	0.103	0.367	−0.172	0.096	0.375	**−0.638**	−0.183	−0.006
T7	−0.104	0.218	0.387	−0.144	0.212	0.394	**−0.530**	−0.042	−0.035
T8	0.072	0.189	0.312	0.008	0.177	0.310	**−0.569**	−0.249	−0.315
T9	0.190	0.192	0.341	0.160	0.156	0.330	−0.158	−0.592	−0.230
T10	0.298	0.184	0.278	0.272	0.162	0.274	−0.205	−0.375	−0.040
T11	0.058	0.245	0.244	0.012	0.247	0.244	−0.266	−0.182	−0.099
T12	0.009	0.249	0.338	−0.049	0.257	0.336	−0.207	0.026	−0.381
T	−0.253	−0.036	0.208	−0.318	−0.035	0.215	**−0.647**	−0.154	−0.124
Ta	−0.253	−0.036	0.208	−0.318	−0.035	0.215	**−0.647**	−0.154	−0.124
	Lag 9								
T1	−0.416	0.179	0.421	−0.471	0.181	0.415	−0.414	−0.060	−0.174
T2	−0.364	0.259	0.469	−0.432	0.257	0.466	−0.461	−0.090	−0.070
T3	−0.265	0.351	0.529	−0.347	0.347	0.530	−0.500	−0.084	−0.058
T4	−0.072	0.439	0.558	−0.151	0.430	0.563	−0.485	−0.140	0.097
T5	−0.016	0.274	0.471	−0.099	0.258	0.478	−0.389	−0.248	0.062
T6	−0.004	0.284	0.485	−0.100	0.269	0.492	−0.508	−0.202	0.112
T7	−0.012	0.138	0.154	−0.112	0.141	0.165	**−0.609**	0.014	0.096
T8	−0.052	0.029	0.105	−0.128	0.024	0.121	**−0.615**	−0.163	0.451
T9	0.312	−0.006	−0.028	0.262	−0.021	−0.020	−0.354	−0.153	0.432
T10	0.161	0.061	0.038	0.100	0.043	0.059	**−0.560**	−0.127	0.419
T11	−0.073	0.229	0.140	−0.085	0.234	0.159	**−0.688**	0.185	0.356
T12	−0.120	0.224	0.190	−0.136	0.244	0.208	**−0.690**	0.290	0.219
T	−0.182	0.188	0.387	−0.252	0.178	0.393	**−0.645**	−0.205	0.102
Ta	−0.182	0.188	0.387	−0.252	0.178	0.393	**−0.645**	−0.205	0.102
	Lag 10								
T1	-	**0.554**	**0.718**	-	**0.531**	**0.725**	-	−0.026	0.084
T2	-	**0.670**	**0.786**	-	**0.646**	**0.792**	-	−0.029	0.140
T3	-	**0.712**	**0.795**	-	**0.689**	**0.800**	-	−0.057	0.147
T4	-	**0.763**	**0.726**	-	**0.743**	**0.734**	-	0.020	0.202
T5	-	**0.731**	**0.706**	-	**0.708**	**0.715**	-	−0.058	0.152
T6	-	**0.729**	**0.711**	-	**0.707**	**0.724**	-	−0.079	0.255
T7	-	**0.514**	0.471	-	**0.508**	**0.493**	-	−0.013	0.415
T8	-	**0.562**	0.512	-	**0.553**	**0.527**	-	0.052	0.327
T9	-	0.390	0.248	-	0.394	0.270	-	0.302	0.487
T10	-	**0.514**	0.348	-	**0.524**	0.364	-	0.317	0.327
T11	-	0.315	0.476	-	0.326	**0.479**	-	0.402	0.015
T12	-	0.157	0.414	-	0.162	0.411	-	0.121	−0.140
T	-	**0.657**	**0.693**	-	**0.643**	**0.707**	-	0.051	0.291
Ta	-	**0.657**	**0.693**	-	**0.643**	**0.707**	-	0.051	0.291

Note: temperature variables: T1–T12—water temperature at stations 1–3 of the Kola section at 50–200 m depth from January to December, respectively, T—averaged year water temperature, Ta—temperature anomaly. Crab indices: Ab—abundance, Bm—biomass, W—weight. Age groups: 1—aged 6–9, 2—aged 10–14, 3—aged 15–19. Bold font indicates significant *p*-values confirmed by the Benjamini–Hochberg procedure. Correlation coefficients for the 0–3, 5–7, and 11–19 lag datasets were insignificant.

## Data Availability

The data presented in this study are available on request from the corresponding author (the data are not publicly available due to privacy restrictions).
